# Alloreactive Regulatory T Cells Allow the Generation of Mixed Chimerism and Transplant Tolerance

**DOI:** 10.3389/fimmu.2015.00596

**Published:** 2015-11-23

**Authors:** Paulina Ruiz, Paula Maldonado, Yessia Hidalgo, Daniela Sauma, Mario Rosemblatt, Maria Rosa Bono

**Affiliations:** ^1^Departmento de Biología, Facultad de Ciencias, Universidad de Chile, Santiago, Chile; ^2^Departamento de Tecnología Médica, Facultad de Medicina, Universidad de Chile, Santiago, Chile; ^3^Fundación Ciencia y Vida, Santiago, Chile; ^4^Facultad de Ciencias Biológicas, Universidad Andres Bello, Santiago, Chile

**Keywords:** alloreactive regulatory T cells, mixed chimerism, transplant tolerance, organ transplantation, non-myeloablative conditioning

## Abstract

The induction of donor-specific transplant tolerance is one of the main goals of modern immunology. Establishment of a mixed chimerism state in the transplant recipient has proven to be a suitable strategy for the induction of long-term allograft tolerance; however, current experimental recipient preconditioning protocols have many side effects, and are not feasible for use in future therapies. In order to improve the current mixed chimerism induction protocols, we developed a non-myeloablative bone-marrow transplant (NM-BMT) protocol using retinoic acid (RA)-induced alloantigen-specific Tregs, clinically available immunosuppressive drugs, and lower doses of irradiation. We demonstrate that RA-induced alloantigen-specific Tregs in addition to a NM-BMT protocol generates stable mixed chimerism and induces tolerance to allogeneic secondary skin allografts in mice. Therefore, the establishment of mixed chimerism through the use of donor-specific Tregs rather than non-specific immunosuppression could have a potential use in organ transplantation.

## Introduction

Achieving transplant tolerance as a means to improve the outcome of organ transplantation is one of the main challenges of modern immunology. Current immunosuppressive drug regimens succeed in preventing acute organ rejection; however, they fail to induce long-term operational tolerance and cannot prevent chronic rejection (1–3).

The generation of mixed chimerism has become an attractive alternative to achieving the induction of long-term tolerance to allografts (4, 5). Mixed chimerism is defined as the coexistence of donor and recipient hematopoietic cell precursors in a pre-conditioned allogeneic bone-marrow transplant (BMT) recipient (4–6). There is robust evidence showing that the induction of mixed chimerism allows the generation of alloantigen-specific tolerance in animal models (7–11) and humans (12–15).

However, the generation of mixed chimerism requires myeloablative preconditioning protocols that can be risky and ethically unfeasible for application on patients who are eligible for a transplant. Different types of regimens have been designed to reduce the myeloablation necessary for the establishment of mixed chimerism (16, 17). Many of them use non-myeloablative doses of total body irradiation (TBI) (18, 19), either alone or in combination with the administration of immunosuppressive drugs (20), depleting antibodies (21–23), and/or co-stimulation blockers (7, 24). The inhibition of CD40/CD40L pathway is critical to induce mixed chimerism without T cell depletion (7, 25, 26). Nevertheless, CD40/CD40L pathway inhibition has been associated with thromboembolic complications (27, 28), and its approval for clinical use remains doubtful.

The use of cellular therapy, which mediates immune tolerance in an antigen-specific manner, may reduce the degree of immunosuppression or co-stimulation blockade necessary for the establishment of mixed chimerism to a minimum. Regulatory T cells have been widely studied for their potential use in this type of therapies. These cells prevent autoimmune and inflammatory diseases, regulate immune responses against viral, bacterial, or parasitic infections, and can also restrain responses directed toward tumors or transplanted tissues (29–33). Two different types of Tregs have been described; natural Tregs CD4 + CD25 + Foxp3 + (nTregs), which are generated in the thymus, and induced Tregs CD4 + CD25 + Foxp3 + (iTregs), which develop in the periphery from naïve CD4 + T cells. Both subsets of Tregs mediate their suppression in an antigen-specific manner (34).

Regulatory T cells have been used in several different cell therapies to treat experimental pathological models. These cells have been shown to prevent graft-versus-host disease (35), delay allograft rejection (36–38), and also provide protection for autoimmune disease therapies (39). Most of the protocols expand the existing pool of Treg cells, through the use of CD3 + CD28+ beads to obtain sufficient number of Treg to use in cellular therapy (40, 41). However, the polyclonal expansion of Tregs could generate a pool of cells with a wide spectrum of specificities.

Our group (41, 42) and others (35) have previously demonstrated the role of retinoic acid (RA) in enhancing the differentiation of CD4 + T cell to iTregs (42, 44–46). We developed a protocol to generate iTregs from naïve T cells in presence of allogeneic CD11c-enriched antigen-presenting cells (APCs), RA, and TGF-β. These, allogeneic RA-iTregs express several immunosuppressive molecules on the surface, have a potent suppressive capacity, show a stable phenotype, and avoid alloantigen-specific skin transplant rejection (42).

In this study, we sought to determine whether allogeneic RA-iTregs could improve current preconditioning protocols of BMT to establish mixed chimerism and induce donor-specific tolerance (25, 43). To test this, we generated a new ­non-­myeloablative bone-marrow transplant (NM-BMT) protocol using allogeneic RA-iTregs, together with Rapamycin, Abatacept (CTLA4Ig), combined with low-dose TBI. This protocol allows the generation of stable mixed chimerism that induces tolerance to allogeneic skin grafts without the administration of additional immunosuppressive drugs.

## Materials and Methods

### Animals

Six to 12-week-old C57BL/6 (Donor, H-2b), BALB/c, and B10.BR (Recipients, H-2d and H-2k, respectively) mice were purchased from The Jackson Laboratory (Bar Harbor, ME, USA). Animals were kept in an animal facility under standard housing guidelines. The animal work was carried out under the institutional regulation of Fundacion Ciencia & Vida and was locally approved by an ethical review committee.

### Antibodies and Reagents

The characterization of Tregs and the analysis of different populations in blood from NM-BMT mice were performed by flow cytometry using the following antibodies: anti-H2K^b^ FITC (AF6.88.6), anti-H2K^d^ PE (SF1-1.1), anti GITR FITC (DTA-1), anti-CD19 APC-H7 (1D3), and anti-CD25 FITC (7D4) from BD Pharmingen (Franklin Lakes, NJ, USA); anti-CD4 FITC (RM4-5), anti-CD4 PE-Cy5 (RM4-5), anti-CD25 PE (PC61.5), anti-CD25 APC (PC61, 5), anti-CD8a PE-Cy7 (53-6.7), anti-GR1 PE-Cy7 (GL7), anti-FoxP3 PE-Cy7 (FJK-16s), anti-CD4 APC (6K1.5), anti-CD3 APC (17A2), and anti-CD16/32 (2.4G2) from eBioscience (San Diego, CA, USA); and anti-CD4 PE (RM4-5) and anti-F4/80 APC-Cy7 (BM8) from BioLegend (San Diego, CA, USA).

Recombinant human TGF-β1 was purchased from eBioscience (San Diego, CA, USA), mouse recombinant IL-2 from R&D System (Minneapolis, MN, USA), and RA from Sigma-Aldrich (St Louis, MO, USA).

### Non-Myeloablative Bone-Marrow Transplant

BALB/c mice were irradiated with high-energy photons using the Oncor Impression PlusLinear Accelerator (Siemens, Munich, Germany). Mice received 3 Gy of TBI 1 day before BMT (day −1).

Groups of age-matched recipient mice received three doses of Rapamycin (LC Laboratories, Woburn, MA, USA) (5 mg/kg) at day −1, 0, and 2 (25). At day 0, BALB/c mice received a dose of 20 × 10^6^ bone-marrow (BM) cells from C57BL/6 mice with or without 2.2 × 10^6^ of RA-iTregs generated from BALB/c mice. On day 2, recipient mice received a single dose (24 mg/kg) of Abatacept (CTLA4Ig) (Orencia, Bristol-Myers Squibb Pharmaceuticals, Princeton, NJ, USA).

### Generation and Isolation of Retinoic Acid-Induced Regulatory T Cells

Alloantigen-specific RA-iTregs were generated following published protocols (43, 44). Briefly, RA-iTregs were generated by stimulating naive T cells from BALB/c mice (sorted as CD4 + CD25−CD62LhiCD44low) in the presence of C57BL/6 APCs plus RA (10 nM), TGF-β (2 ng/ml), and IL-2 (10 ng/ml). After 6 days of culture, RA-iTregs were further purified by cell sorter as CD4+ CD25hi cells using a FACSAria II Cell Sorter (BD Bioscience, San Diego, CA, USA). The purity of the RA-iTregs preparation was always over 95%. Polyclonal RA-iTregs were generated by stimulating naive T cells from BALB/c mice in the presence of BALB/c APCs plus anti-CD3 mAb RA (10 nM), TGF-beta (2 ng/ml), and IL-2 (10 ng/ml).

The phenotype of RA-iTreg was determined by flow cytometry (FACS Canto II, BD Bioscience, San Diego, CA, USA). The analysis was carried out using the FlowJo version 8.7 software (Tree Star Inc. Ashland, OR, USA). Intracellular Foxp3 staining was done using the Foxp3 staining Kit (eBioscience, San Diego, CA, USA) following the manufacturer’s instructions.

### Skin Transplantation

For skin transplant experiments, we used C57BL/6 (H-2^b^) mice as skin donors and BALB/c (H-2^d^) mice as transplant recipients. Skin transplantation was performed 4 weeks post BM transplant. The recipient mice were anesthetized with 3% XEVORane (Abbot, Buenos Aires, IL, Argentina). Full thickness tail skin from mismatched C57BL/6 mice of ~1 cm × 1 cm was transplanted on the left lateral flank of recipient mice. Mice were bandaged for 10 days, and the acceptance or rejection of the graft was analyzed by macroscopic visual inspections at short time intervals. Rejection was determined when transplanted skin formed a dry scar (44, 45, 49, 50).

### Chimerism Analysis by Flow Cytometry

To evaluate mixed chimerism, we measured the presence of donor MHC-I positive cells in blood and expressed it as the percentage of donor MHC-I positive cells over total leukocyte lineage. The percentages of the different donor populations were determined by labeling the cells with anti-CD3, anti-CD4, anti-CD8, anti-CD19, anti-GR1, and anti-F4/80 in combination with donor MHC-I antibody. We performed the analyses on 3, 4, 7, 10, 13, and 16 weeks after NM-BMT.

### ELISPOT Assays

Splenocytes were recovered from BALB/c recipients 110 to 120 days after skin graft transplantation. These cells were re-stimulated for 24 h with previously irradiated (30 Gy) allogeneic (C57BL/6) or syngeneic (BALB/c) splenocytes. To measure the frequency of IFN-γ, IL-4-, and IL-17-producing cells, we used the mouse ELISPOT Ready-SET-go Kit (eBioscience San Diego, CA, USA) according to the manufacturer’s instructions. The frequencies of cytokine-secreting cells were expressed as the number of cytokine-producing cells per 1 × 10^5^ responder cells. All assays were done in triplicate.

### *In vitro* MLR Suppression Assay

CD4 + CD25− responder T cells (5 × 10^4^) were sorted from BALB/c mice and labeled with CellTrace Violet dye (Life Technologies). As stimulator cells, we used 1 × 10^5^ previously irradiated splenocytes (30 Gy) from C57BL/6 (allogeneic), B10.BR (third-party) or BALB/c (syngeneic) mice. The responder and stimulator cells were co-cultured at different ratios with CMTMR-labeled RA-iTreg or with anti-CD3 mAb. After 5 days, CellTrace Violet dilution in responder cells was analyzed by flow cytometry.

### Statistical Analysis

Statistical analyzes were performed using Prism program for Macintosh, version 4.0b (GraphPad Software Inc.). In order to establish the statistical distribution of the results, we used the Shapiro–Wilk normality test. Depending on the sampling distribution, a non-parametric Mann–Whitney test, a parametric Student’s *t*-test, or one-way ANOVA was used. The graft survival curves are shown using a Kaplan–Meier analysis and the statistical significance was estimated by LogRank test. *p*-values ≤0.05 were considered statically significant. The data are presented as mean ± error standard (SEM).

## Results

### Donor-Specific RA-iTreg Together with a NM-BMT Protocol Induces Stable Mixed Chimerism

**Figure 1:**
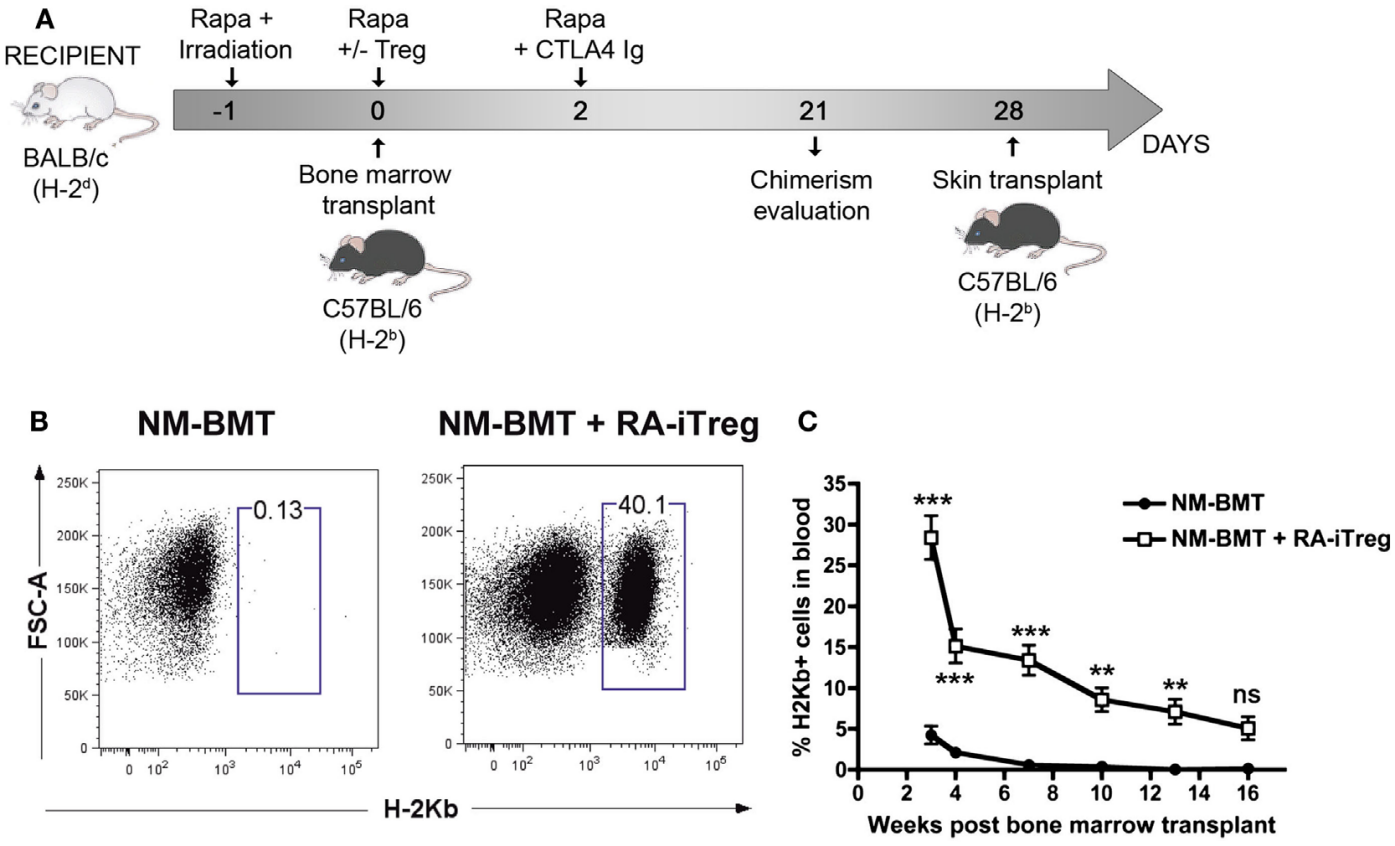
**Alloantigen-specific RA-iTregs allow the generation of stable mixed chimerism in NM-BMT mice**. **(A)** Schematic representation of NM-BMT regimen. BALB/c mice received NM-BMT regimens that consisted in the administration of low doses of irradiation (3 Gy of TBI at day −1), together with co-stimulation blockade with Abatacept (CTLA4Ig) (24 mg/kg at day 2), and three doses of Rapamycin (5 mg/kg at days −1, 0, and 2). At day 0, mice received 20 × 10^6^ bone-marrow cells from C57BL/6 mice and 2.2 × 10^6^ BALB/c RA-iTreg cells or PBS. **(B)** Chimerism at 3 weeks post-transplantation was analyzed by the presence of H-2K^b^ positive cells in blood. **(C)** Kinetics of mixed chimerism, analyzed as the presence of H-2K^b^ positive cells in peripheral blood samples from NM-BMT mice treated with (□) or without (●) RA-iTregs. The statistical one-way ANOVA test was used. Bars represent the SE. The data represent seven independent experiments. Rapa, Rapamycin; Aba, Abatacept; RA-iTregs, retinoic acid-induced Tregs cells; BMT: Bone-marrow transplantation. ***p < 0.0001; **p < 0.001; *p < 0.01.

In a previous study, we generated donor-specific retinoic acid-induced Tregs (RA-iTregs) and used these cells to prevent skin allograft rejection in an immunodeficient Rag1−/− mouse model (42). However, it remained to be established whether these donor-specific RA-iTregs were also able to generate mixed chimerism and allow transplantation tolerance in immunocompetent mice. To test this, we designed a NM-BMT protocol that may be translated to clinical use. This NM-BMT protocol consists of 3 Gy TBI (at day −1), three doses of Rapamycin (5 mg/kg at day −1, 0, and 2), and a single dose of Abatacept (CTLA4Ig) (24 mg/kg at day +2). Additionally, some of the recipient mice received a single dose of RA-iTregs at day 0 (see diagram on Figure 1A).

Next, we sought to study the ability of allospecific Tregs to induce mixed chimerism. For this, we stimulated naive T cells obtained from BALB/c mice (H-2^d^) with allogeneic APCs from C57BL/6 mice (H-2^b^) in the presence of TGF-β, RA, and IL-2. Figure S1A in Supplementary Material shows that RA-iTregs generated after 6 days of culture express several markers related to Treg function such as CTLA4, LAG3, CD73, CD39, GARP, CD103, and GITR. Then, we tested whether these RA-iTregs could specifically inhibit the allogeneic response. As shown in Figure S1B in Supplementary Material, RA-iTregs specifically inhibited the proliferation of T cells cultured with C57BL/6 APCs but not cultures done with third-party APCs (B10.BR). Moreover, allogeneic RA-iTregs are more efficient than polyclonal RA-iTregs at inhibiting the proliferation of T cells in mixed leukocyte reactions (Figure S2 in Supplementary Material).

To study the ability of these allospecific RA-iTregs in promoting mixed chimerism, donor H-2K^b^ cells in peripheral blood samples of NM-BMT mice were assessed by flow cytometry. As shown in Figure 1B, mice receiving RA-iTregs presented donor-derived (H-2K^b+^) cells compared with the group that did not receive RA-iTregs. This result demonstrates that RA-iTregs can generate mixed chimerism in our NM-BMT protocol.

To evaluate the stability of mixed chimerism generated by a single dose of RA-iTregs, we determined over time the presence of H-2K^b^ cells in peripheral blood of mice. Figure 1C shows that the group that did not receive RA-iTregs presented lower levels of chimerism, disappearing by 7 weeks after BMT. By contrast, mice treated with RA-iTregs displayed a statistically significant percentage of mixed chimerism up to 16 weeks post BMT, indicating that a single dose of RA-iTregs is sufficient to induce a stable mixed chimerism.

Next, we evaluated reconstitution of the chimera with multi-lineage donor leukocytes. For this, we analyzed CD4+ and CD8+ T cell subsets, B lymphocytes (CD19+), monocytes (F4/80+), and granulocytes (GR-1+) by flow cytometry in blood samples at different time points post NM-BMT (Figures S3A,B in Supplementary Material). We observed increasing percentages of T lymphocytes, with a similar contribution from CD4+ and CD8+ T cells. The levels of B cells were maintained over time and represented a substantial percentage of total chimerism. Furthermore, we observed granulocyte and monocyte reconstitution. However, in contrast to lymphoid cells, myeloid cells showed a decrease over time. Therefore, we observed that the mice subjected to NM-BMT in combination with RA-iTregs repopulated all cellular hematopoietic lineages from donor precursors.

### RA-iTregs Together with NM-BMT Prolong Allograft Survival

**Figure 2:**
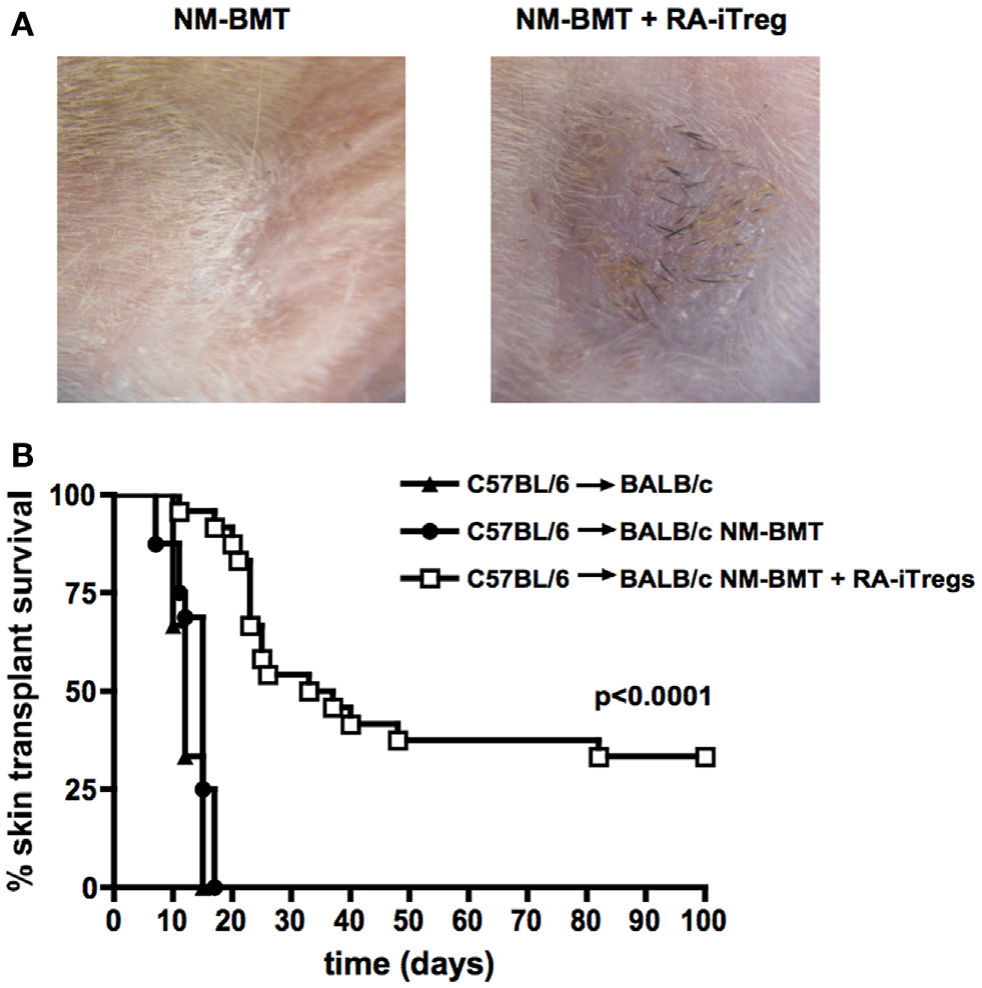
**NM-BMT mice treated with RA-iTregs have a prolonged allogeneic skin graft survival**. Skin transplants were performed using C57BL/6 mice as donors of tail skin in untreated BALB/c mice (BALB/c) (▲) (*n* = 3); bone-marrow-transplanted BALB/c mice (BALB/c NM-BMT) (●) (*n* = 13); or bone-marrow-transplanted BALB/c plus RA-iTregs (BALB/c NM-BMT + RA-iTreg) (□) (*n* = 17) as receptors. **(A)** Image of skin transplant at day 50 post-transplant. **(B)** Kaplan–Meier survival analysis of skin allograft transplantation for 100 days post-transplant. LogRank Statistical test (comparison of survival curves) was performed. *p*-value <0.05. The results represent four independent experiments.

To determine whether chimeras obtained with RA-iTregs induce tolerance mechanisms in BALB/c recipient mice, we performed allogeneic skin grafts using C57BL/6 mice as skin donors. Transplants were done 4 weeks after the initiation of the non-myeloablative protocol. We separated recipient mice into three groups: non-treated mice, NM-BMT mice, and RA-iTreg treated NM-BMT mice. Figure 2A shows the presence of skin and black hair at the site of the transplant 100 days after skin transplant in RA-iTreg-treated mice. Mice that received only NM-BMT quickly rejected the allograft as shown by the scar at the site of operation. Figure 2B shows that Treg-treated NM-BMT mice showed a significant increase (*p* < 0.0001) in the survival of the transplanted skin, exceeding 100 days post-transplant, without the need for any additional immunosuppression. Therefore, mixed chimeras generated with the combination of NM-BMT and RA-iTregs induce tolerance mechanisms that allow the acceptance of allogeneic skin grafts.

### RA-iTregs Together with NM-BMT Inhibit Allogeneic-Induced Th1 Cellular Responses

**Figure 3:**
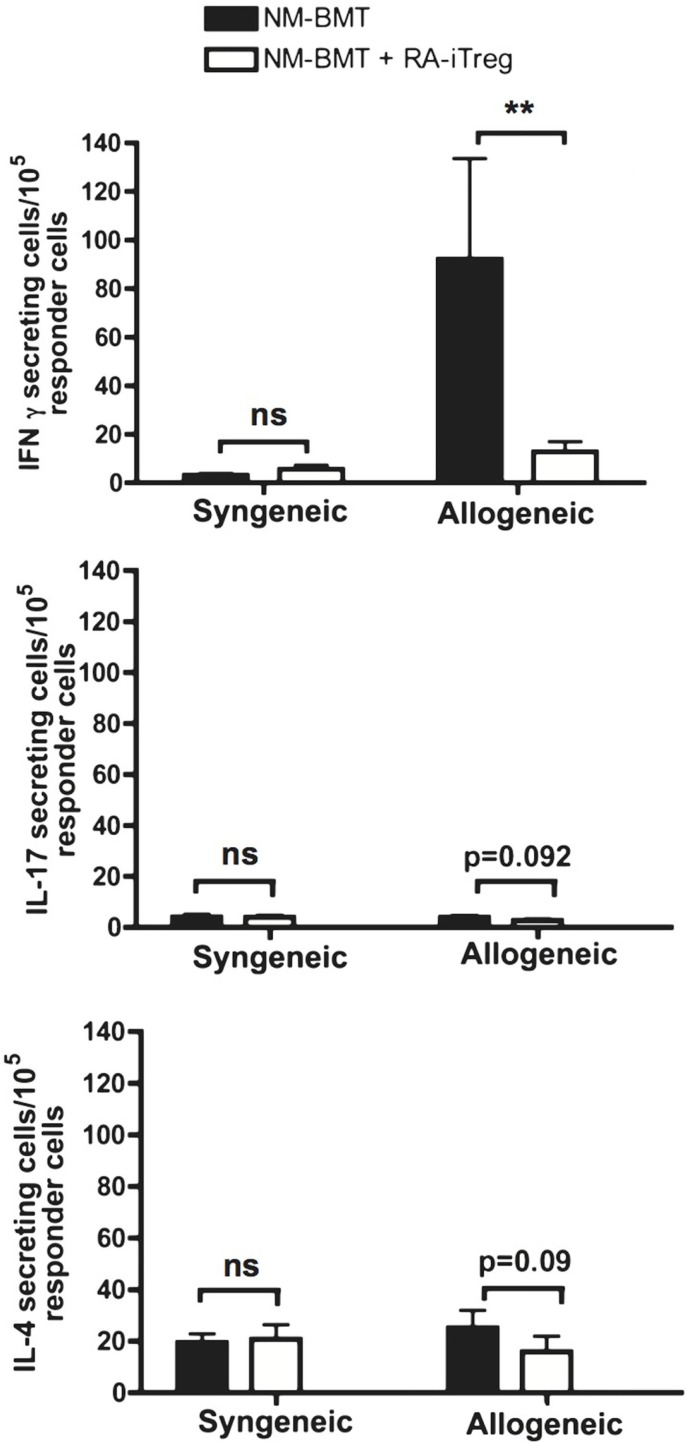
**Th1 allogeneic immune response is inhibited in NM-BMT mice that received RA-iTregs**. One hundred days after skin transplant, splenocytes from NM-BMT mice treated with or without RA-iTregs were co-cultured with APC from BALB/c (syngeneic) or C57BL/6 (allogeneic) mice. IFN-γ, IL-17, and IL-4 cytokine secretion was measured by ELISPOT. Bars show the SE. The Mann–Whitney–Wilcoxon statistical test for non-parametric distributions was used. The results represent two independent experiments.

The main factors involved in organ rejection are cellular immune responses mediated by Th1 and Th17 cells (46, 47). It has been reported that the induction of mixed chimerism promotes Th2 responses that favor alloimmune tolerance (53). Thus, we investigated by ELISPOT assays the type of alloimmune response produced by RA-iTregs-treated NM-BMT mice at the end of the skin transplant protocol. For this, splenocytes from transplanted mice were co-cultured for 24 h with syngeneic or allogeneic splenocytes on pre-coated ELISPOT plates to analyze IFN-γ-, IL-4, and IL-17 secreting cells. We used as controls splenocytes from NM-BMT mice that rejected the skin transplant. As shown in Figure 3, we observed a strong Th1 response, as measured by IFN-γ secretion, only when splenocytes from NM-BMT are stimulated with allogeneic splenocytes. We observed that RA-iTregs-treated NM-BMT had a significant decrease in IFN-γ secreting cells comparable to the level found in syngeneic cocultures (*p* = 0.008). We did not see any significant IL-4 or Il-17 cytokine secretion in all conditions, indicating that Th2 and Th17 cellular responses do not mediate allograft rejection in our setting. These results suggest that peripheral tolerance mechanisms induced in RA-iTreg-treated NM-BMT mice effectively abolish Th1 alloimmune responses.

### Chimeric Mice Exhibit Donor-Derived Regulatory T Cells

**Figure 4:**
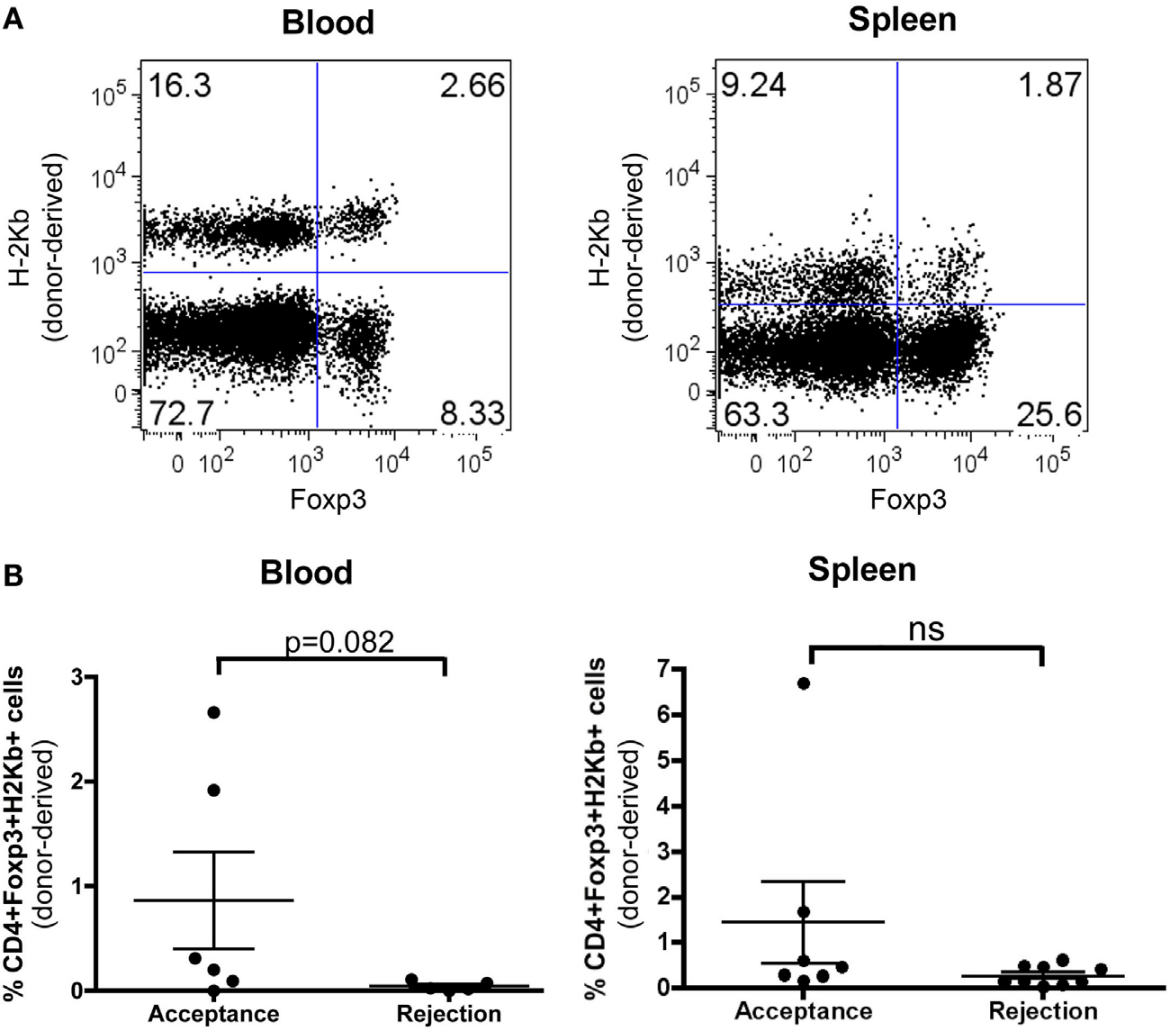
***De novo* generation of donor Tregs in NM-BMT mice receiving RA-iTregs**. **(A)** One hundred days after skin transplant, donor-derived Tregs were analyzed in peripheral blood and spleen in NM-BMT mice treated with RA-iTregs. **(B)** Percentage of CD4 + Foxp3 + H-2K^b+^ in peripheral blood and spleen in mice that accept (*n* = 6) or reject (*n* = 7) the allogeneic skin transplant. Bars represent the SE. The Mann–Whitney–Wilcoxon statistical test was used. The results represent two independent experiments.

To study the mechanism of tolerance induction in RA-iTreg-treated NM-BMT mice, we evaluated the presence of CD4 + CD25 + FoxP3+ Tregs derived from donor precursors cells (H-2^b^) in these mice. As shown in Figure 4, 100 days after skin transplant, we found donor-derived H-2^b^ + CD4 + CD25 + FoxP3+ Tregs in blood and spleen of NM-BMT mice treated with RA-iTregs. When we correlated the presence of donor Tregs with skin transplant acceptance or rejection (Figure 4B), we observed that some mice that accepted allogeneic skin transplant presented donor Tregs in spleen and blood. However, we could not detect donor Tregs in blood and spleen in mice that rejected allogeneic skin.

## Discussion

In this work, we developed a protocol using RA-iTregs to induce mixed chimerism. Here, we combine RA-iTregs with a NM-BMT avoiding the need of CD40/CD40L signaling inhibition. This NM-BMT protocol consisted of a low dose of TBI (3 Gy), three doses of the immunosuppressive drug Rapamycin, and one dose of co-stimulation blockade using Abatacept (CTLA4Ig). We demonstrate that the use of RA-iTregs with this non-myeloablative preconditioning setting promotes mixed chimerism and significantly extends the survival of allogeneic skin graft.

CD4 + CD25 + Foxp3+ Tregs have been used previously to generate mixed chimerism and alloimmune tolerance in two different reports (25, 43). Joffre’s group induced mixed chimerism by using donor-specific Tregs obtained through the expansion of splenic CD4 + CD25 + Foxp3+ cells with allogeneic dendritic cells and IL-2 in combination with 5 Gy TBI (43). Despite the positive results they obtained, the use of high-dose irradiation is critical and limits future application in humans (48, 49). In the second report, the authors tested different types of polyclonal Tregs: nTregs, iTregs, and FoxP3 transduced Tregs, in their ability to induce mixed chimerism (25). These authors induced mixed chimerism by using these Tregs in combination with Rapamycin, co-stimulation blockade with Abatacept, and anti-CD154 as non-myeloablative preconditioning, thereby avoiding the use of radiation. This work demonstrates that natural or induced Tregs allow the generation of mixed chimerism and transplantation tolerance without cytoreductive conditioning (25). Although these results are promising, translation into clinical use in humans faces two problems; first, the use of anti-CD154 is restricted to experimental procedures only, and second, the use of polyclonal Tregs may inhibit immune responses against infections or may cause higher rates of cancer. Here, we sought to improve these preconditioning protocols so that it could be translated into clinical application in humans. The protocol we developed is donor specific and avoids the use of anti-CD154, thus, diminishing the risks associated with its use and allowing its future translation to human trials (27).

The goal of transplant immunology is to provide specific-donor immunosuppression, and this specificity could be achieved by alloantigen-specific Tregs. Here, we show that RA-iTregs express immunosuppressive molecules and specifically inhibit the proliferation of effector T cells that recognized the same alloantigen. The latter observation is in agreement with previous work from our group (42, 44), where we demonstrated that RA-iTreg cells showed alloantigen-specific immunosuppressive capacity in a skin allograft model in immunodeficient mice. In support of this, work by Joffre’s group show selective grafting of allogeneic bone marrow from the specificity of Tregs, whereas bone marrow from a third strain was rejected (43, 48). Moreover, in a previous report, we have shown that RA-iTreg cells are stable in time when adoptively transferred and present an unbiased homing capability (42). Thus, alloantigen-specific Tregs may establish specific tolerance while maintaining immune responsiveness to foreign antigens.

A recent article published by Wekerle’s group reports that rapamycin and CTLA4Ig synergize to induce stable mixed chimerism (50). Their strategy to induce mixed chimerism is very similar to ours except that they give mice an extra dose of CTLA4Ig and they use 2 Gy of TBI. Importantly, they did not use Tregs to induce chimerism. In our hands, we obtain stable mixed chimerism only when we add RA-iTregs. The differences between Wekerle’s and our results may be due, in part, to the combination of donor-recipient mouse strains used in both studies. Although we achieved some chimerism without Tregs, it was only transient since it was lost by 7 weeks. On the other hand, Wekerle and collaborators induce donor-specific tolerance to heart allografts but they do not succeed with skin allograft as we did, which is considered to be more stringent than the heart transplant. Here, we developed a powerful long-term operational tolerance protocol to donor-specific skin allograft, surpassing minor and major antigens barriers to produce mixed chimerism induction.

The protocol we used in this work to induce mixed chimerism allows the reconstitution of all hematopoietic cell populations frequently found in peripheral blood from the donor. The percentage of lymphocytes remains stable or increases over time while macrophages and granulocytes tend to decrease. The reasons why myeloid cells change over time have not been elucidated; however, the establishment of tolerance has been associated with the presence of donor T cells (51). It has been shown that tolerance induced by mixed chimerism can be reversed by depleting CD4+ T cells (52, 53). Thus, the decrease in the myeloid populations could be an irrelevant factor in the establishment of tolerance although this should not be entirely disregarded. Although we could not achieve a 100% graft survival, here, we demonstrate that mixed chimerism induced by RA-iTregs extends the life of the allogeneic skin transplant. As transplant tolerance correlates with the level of chimerism (54, 55), we could speculate that the percentage of graft survival could be increased if the transplant is performed at earlier times following the BMT.

Mice that received our conditioning protocol of NM-BMT together with RA-iTregs exhibited donor-derived Tregs. These cells were detected in blood and spleen 120 days after the transplant. Previous reports where mixed chimerism was obtained through non-myeloablative preconditioning and depletion of donor CD25+ cells in the transplanted BM, showed no transplant acceptance (56). Moreover, the depletion of donor or recipient Foxp3+ cells following skin and heart transplant induces the loss of allogeneic tolerance mediated by mixed chimerism (57). Although we observed a positive correlation between the presence of donor-derived Tregs and the acceptance of skin transplant, this correlation was not statistically significant. A complete evaluation of this donor Tregs in other tissues of mice that accepted or rejected their allografts are needed in order to determine the origin and contribution of these cells in transplant tolerance.

The translation of this protocol to induce donor-specific tolerance in humans is possible since several protocols to differentiate human CD4+ T cells into Tregs have been described (58). Therefore, the establishment of mixed chimerism through the use of donor-specific Tregs rather than general or non-specific immunosuppression could constitute a reasonable alternative in organ transplantation.

## Author Contributions

PR wrote the paper and performed the research. PM performed the research. YH performed the research. DS wrote the paper. MR wrote the paper. MB designed the research and wrote the paper.

## Conflict of Interest Statement

The authors declare that the research was conducted in the absence of any commercial or financial relationships that could be construed as a potential conflict of interest.

## Abbreviations

APCs, antigen-presenting cells; BMT, bone-marrow transplant; NM-BMT, non-myeloablative bone-marrow transplantation; RA, retinoic acid; RA-iTregs, retinoic acid-inducible regulatory T cells; TBI, total body irradiation; Tregs, regulatory T cells.
