# Supplementary material for: Alloreactive Regulatory T Cells Allow the Generation of Mixed Chimerism and Transplant Tolerance
Source: Front Immunol. 2015 Nov 23;6:596. doi: 10.3389/fimmu.2015.00596 (PMC4655502; doi:10.3389/fimmu.2015.00596)
Supplement: Supplementary file 1 [file Image_1.PDF]

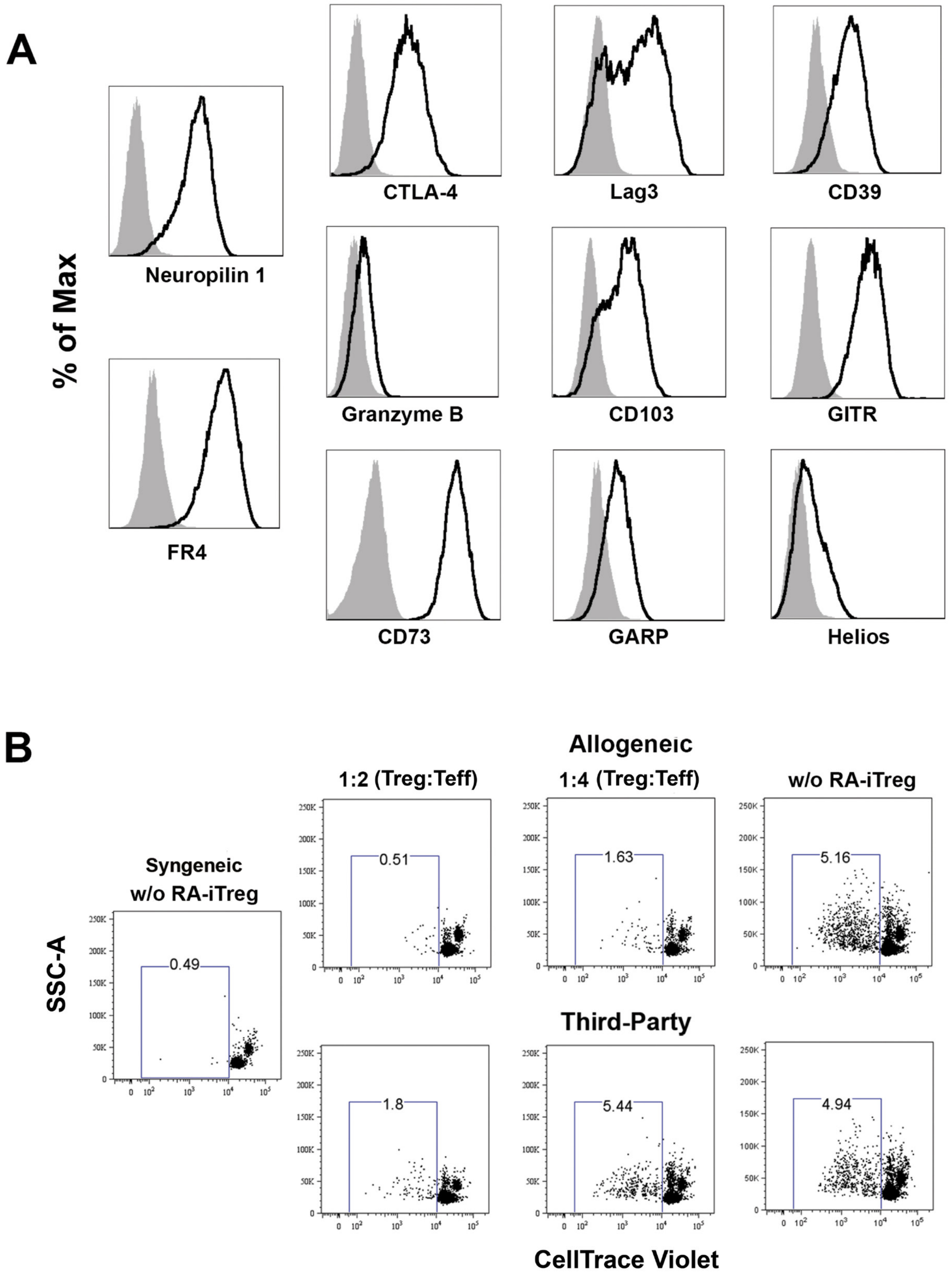

Supplementary Figure 1 | Phenotype and Donor specificity of RA-iTregs.

A. RA-iTregs were generated by the co-culture of naive T cells from BALB/c mice with APC from C57BL/6 mice in presence of IL-2 (10 ng/mL), TGF-β (2 ng/mL) and retinoic acid (10 nM). Grey histograms represent the autofluorescence or secondary antibody control. Representative experiment of three independent experiments.

B. Antigen presenting cells from C57BL/6, BALB/c and B10.BR were co-cultured for five days with cell labeled effector CD4+ T cells from BALB/c mice and different ratios of RA-iTregs (BALB/c against C57BL/6). Proliferation was measured as Violet CellTrace dilution by flow cytometry. The data are representative of three independent experiments.
