# Supplementary material for: Alloreactive Regulatory T Cells Allow the Generation of Mixed Chimerism and Transplant Tolerance
Source: Front Immunol. 2015 Nov 23;6:596. doi: 10.3389/fimmu.2015.00596 (PMC4655502; doi:10.3389/fimmu.2015.00596)
Supplement: Supplementary file 2 [file Image_2.PDF]

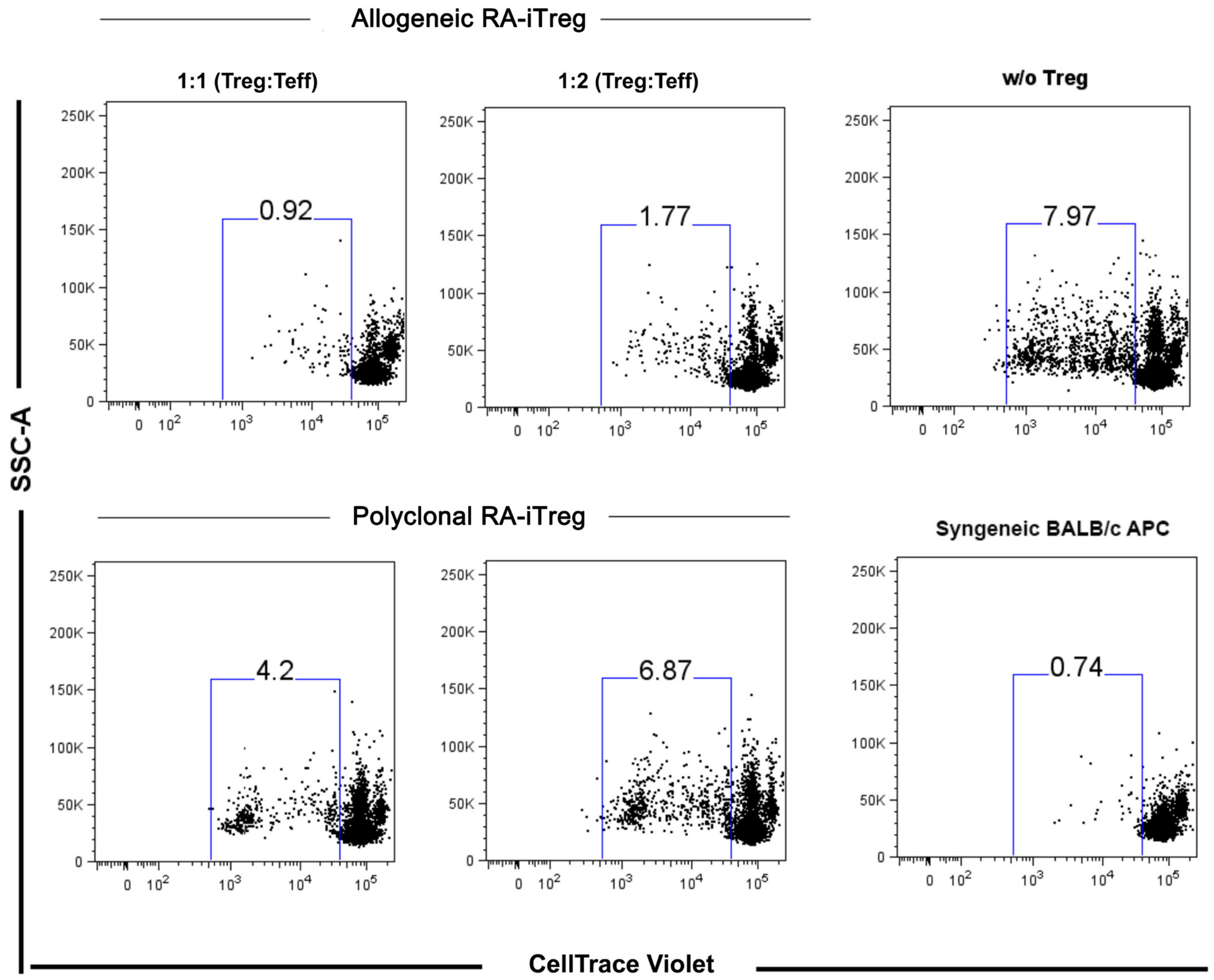

Supplementary Figure 2 | Allogeneic RA-iTregs are more efficient than polyclonal RA-iTregs in suppressing MLR.

Antigen presenting cells from C57BL/6 and BALB/c were co-cultured for five days with cell trace-labeled effector CD4+ T cells from BALB/c mice and different ratios of polyclonal or allogeneic RA-iTregs. Proliferation was measured as Violet CellTrace dilution by flow cytometry. The data are representative of three independent experiments.
