# Supplementary material for: Alloreactive Regulatory T Cells Allow the Generation of Mixed Chimerism and Transplant Tolerance
Source: Front Immunol. 2015 Nov 23;6:596. doi: 10.3389/fimmu.2015.00596 (PMC4655502; doi:10.3389/fimmu.2015.00596)
Supplement: Supplementary file 3 [file Image_3.PDF]

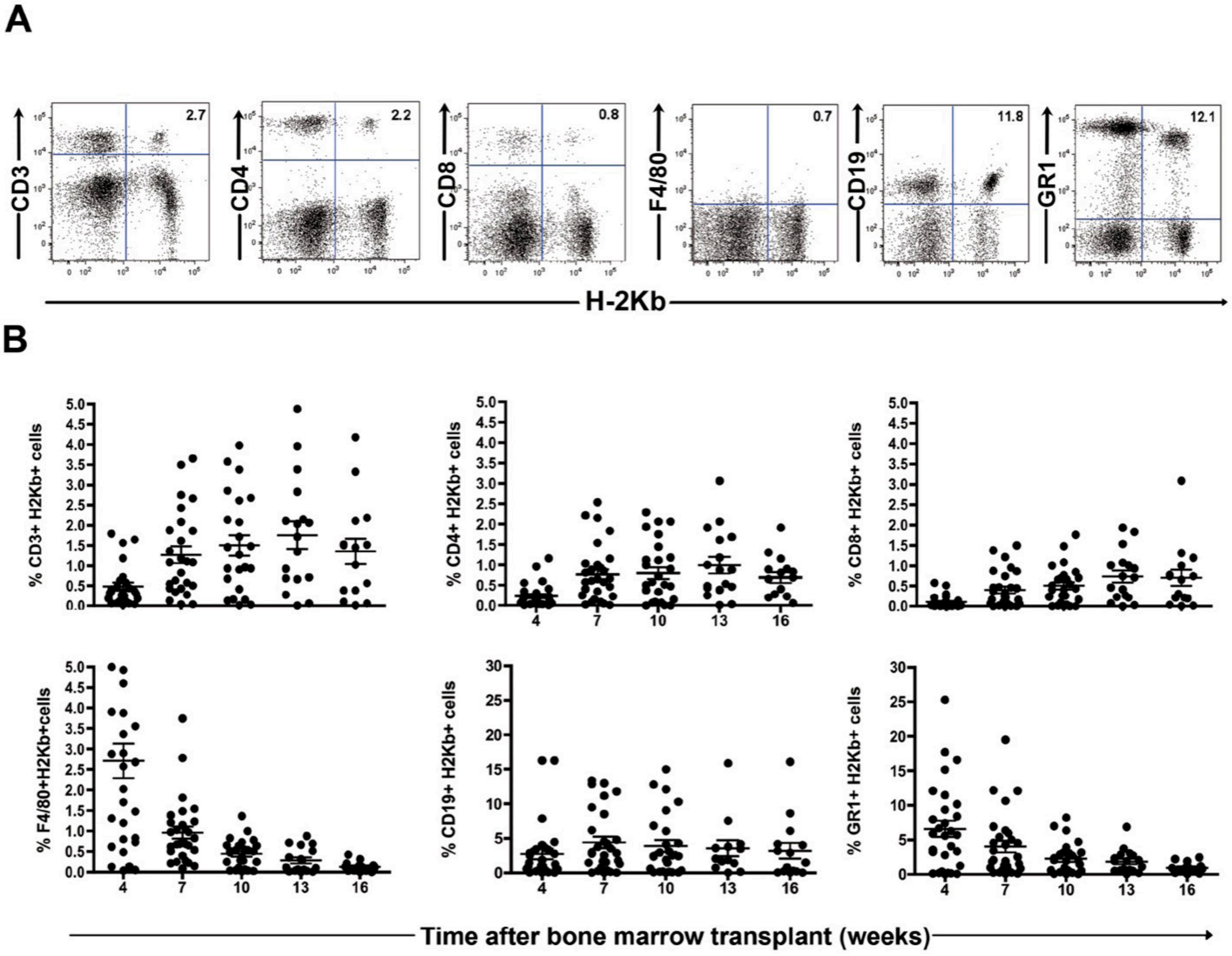

Supplementary Figure 3 | Multi lineage chimerism in NM-BMT mice treated with RA-iTregs. Analysis of different leukocyte populations in blood from NM-BMT mice receiving RA-iTregs. **A**. Representative dot plot analysis of NM-BMT mice receiving RA-iTregs seven weeks after BMT. The results represent five independent experiments. **B**. Quantification of multi-lineage repopulation in BMT mice receiving RA-iTregs. The analysis was performed at different times post-BMT. Bars represent the standard error.
